# Emergency Use of Targeted Osmotic Lysis for the Treatment of a Patient with Aggressive Late-Stage Squamous Cell Carcinoma of the Cervix

**DOI:** 10.3390/curroncol28030196

**Published:** 2021-06-08

**Authors:** Harry J. Gould, Paige R. Miller, Samantha Edenfield, Kelly Jean Sherman, Chad K. Brady, Dennis Paul

**Affiliations:** 1 Department of Neurology, Louisiana State University Health Sciences Center, New Orleans, LA 70112, USA; 2 Oleander Medical Technologies, Baton Rouge, LA 70803, USA; pmiller@oleandermedicaltechnologies.com (P.R.M.); dpaul@lsuhsc.edu (D.P.); 3 Department of Pharmacology and Experimental Therapeutics, Louisiana State University Health Sciences Center, New Orleans, LA 70112, USA; simbra@lsuhsc.edu (S.E.); ksherm@lsuhsc.edu (K.J.S.); 4 Department of Radiology, West Virginia University Medical School, Morgantown, WV 26506, USA; ckbrady@hsc.wvu.edu

**Keywords:** targeted osmotic lysis, advanced-stage cervical cancer, sodium channels, sodium pumps

## Abstract

Upregulation of voltage-gated sodium channels (VGSCs) and Na^+^/K^+^-ATPase (sodium pumps) is common across most malignant carcinomas. Targeted osmotic lysis (TOL) is a developing technology in which the concomitant stimulation of VGSCs and pharmacological blockade of sodium pumps causes rapid selective osmotic lysis of carcinoma cells. This treatment of cervical carcinoma is evidence that TOL is a safe, well-tolerated and effective treatment for aggressive advanced carcinomas that has the potential to extend life without compromising its quality. TOL is likely to have broad application for the treatment of advanced-stage carcinomas.

## 1. Introduction

The simultaneous activation of voltage-gated sodium channels (VGSCs) and pharmacological blockade of sodium/potassium-ATPase (Na^+^/K^+^-ATPase; sodium pumps) with a cardiac glycoside in highly malignant cancer cells causes increased intracellular sodium and sufficient increased oncotic pressure to produce lysis of cells that overexpress VGSCs [1,2]. This “targeted osmotic lysis” (TOL) selectively lyses the most malignant cancer cells while leaving the cells that express VGSCs normally unaffected. To date, TOL has been shown to reduce the size and slow the growth of several forms of advanced carcinomas and increase survival in mice and companion animals without damaging normal tissues or producing discernible adverse effects. Based upon a review of the available information about this novel approach to treating advanced carcinomas, a request was made on behalf of a 46-year-old female by her treating oncologist to allow the emergency use of targeted osmotic lysis, a novel treatment currently under development for treating late-stage carcinomas, because his patient was rapidly losing her battle with stage IIB squamous cell carcinoma of the cervix. The request was based on her extreme level of distress and fatigue as well as significant physical and cognitive limitations. She also experienced intractable pain despite high-dose opioid therapy that, while marginally effective, further exacerbated her cognitive impairment. She had exhausted all currently accepted treatment options and had been excluded from all the available therapeutic trials. The prognosis for her survival was anticipated to be days to two weeks at the time of the request. 

## 2. Case Report

### 2.1. Clinical History

The patient’s cancer was diagnosed in 2016 and initially responded to treatment with concomitant radiation and cisplatin chemotherapy plus adjuvant paclitaxel and 65% of the target dose of carboplatin according to the OUTBACK trial protocol (ANZGOG-0902/GOG-0274/RTOG-1174, ClinicalTrials.gov (accessed on 19 May 2021) identifier NCT01414608), but recurred two years later in the right sidewall of the pelvis as an enhanced soft tissue mass with right lateral extension located within the parametrial region measuring approximately 3.5 cm × 2.2 cm × 2.5 cm that was considered to be unresectable. Despite the patient receiving another 9 cycles of paclitaxel, carboplatin and bevacizumab chemotherapy, the disease continued to progress. The patient was not considered to be a candidate for the Iovance tumor-infiltrating lymphocyte (TIL) therapy and a clinical trial with AGEN1884/2034 because PD-L1 testing of the original tumor was negative. Nonetheless, anti-PD-1 therapy was declined in lieu of three additional cycles of chemotherapy. 

One month later, the mass measured approximately 3.0 cm × 3.3 cm × 3.2 cm. Treatment was initiated with pembrolizumab. Despite four cycles of treatment, the tumor grew to 4.9 cm × 6.7 cm × 6.7 cm. In light of the resistance to pembrolizumab, she was evaluated and denied inclusion in the Iovance clinical trial.

Three months later, a CT scan revealed that her tumor mass had grown to 8.7 cm × 10.7 cm × 8.9 cm and to 12.2 cm × 9.9 cm × 9.4 cm one month later, when her tumor was no longer discernably separable from the presacral space and the sciatic foramen on the right side. The mass involved the obturator muscle, the gluteus muscle and sacral nerve roots S2–S4, the rectum, the adjacent uterus, bowel and urinary bladder. It was understood that, at that point, the patient had exhausted all the available therapeutic options. The patient’s overall condition had deteriorated to the point where her Eastern Cooperative Oncology Group performance status was 4, placing her at a high risk for chemotherapy-related complications; she was no longer considered to be a candidate for clinical trials. The patient was thus left without a clear therapeutic path forward. 

Based upon an evaluation of the available information on TOL [1,2] and given the patient’s extreme distress and that the risk of serious toxicity associated with TOL was lower than exposing the patient to investigational chemotherapeutic agents, an emergency treatment with TOL, a developing technology that uses the overproduction of VGSCs combined with the pharmacological blockade of Na^+^/K^+^-ATPase to induce selective lysis of aggressive carcinomas and metastases [1,2], was requested. The patient was admitted to hospice care with an extremely diminished ability to perform the basic activities of daily living, failure to thrive and intractable pain on a high-dose regimen of morphine, methadone, lorazepam, alprazolam and hydromorphone from a patient-controlled analgesia pump that yielded little relief. 

### 2.2. Preparation, Emergency Treatment and Response

**Figure 1:**
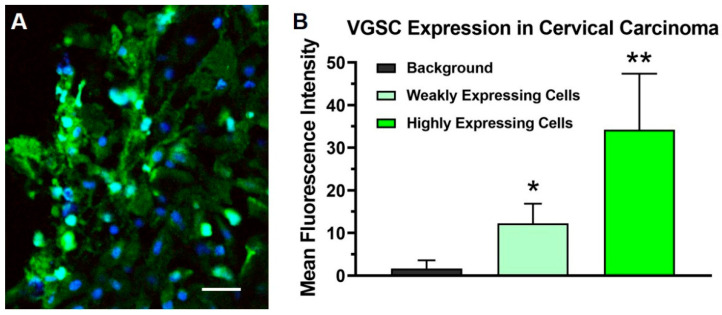
The photomicrographs depict the immunohistochemical labeling of voltage-gated sodium channels (VGSCs) (green) in a biopsy of the stage IIB carcinoma of the cervix obtained from the patient prior to treatment with targeted osmotic lysis (TOL). Sections were incubated and then processed with a goat-anti-rabbit 488 Alexa fluorophore secondary antibody, 1:800 dilution. Nuclei were counterstained with DRAQ5 (blue; dilution 1:600). Twelve images taken with a Leica DMi8 confocal microscope at 40× oil magnification were reviewed for evidence of VGSC labeling. Panel (**A**) depicts a representative portion of the biopsy where many cells, approximately 40% of the total, highly expressed VGSCs. Cellular profiles from three sections were outlined and the mean fluorescence intensity was measured using the ImageJ-FIJI software (imagej.nih.gov; accessed on 19 May 2021) to quantify the sodium channel expression. The histogram (**B**) presents the average mean fluorescence intensity measured from 50 intensely stained and 50 weakly stained cell profiles compared to the background indicative of the level of VGSC expression in highly expressing tumor cells and minimally expressing neoplastic and stromal cells, respectively. A one-way ANOVA was significant (*p* < 0.00001); * = *p* < 0.001 compared to the background; ** = *p* < 0.001 compared to weakly expressing cells (Student’s *t*-test with Scheffe’s adjustment). Calibration bar in A = 25 µm.

**Figure 2:**
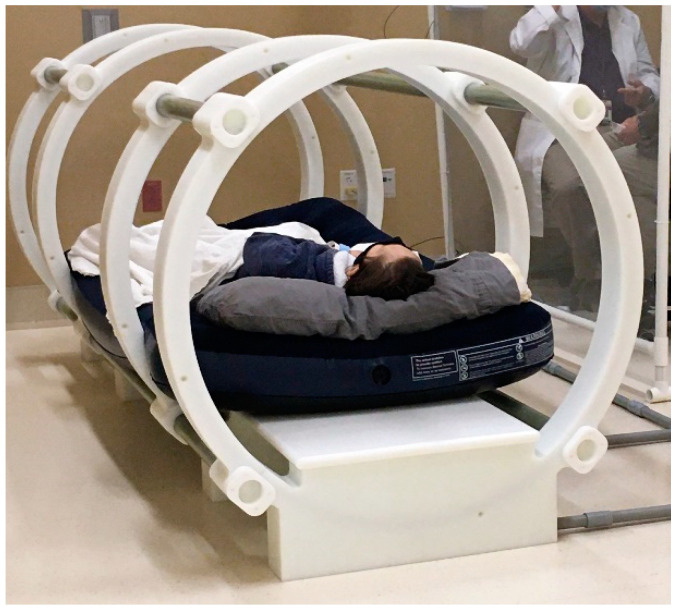
The photograph depicts the patient in the bore of the custom-built coaxial ring device used to deliver the uniform pulsed electric fields necessary for opening the VGSCs in TOL (The Phantom Laboratory, Salem, NY, USA).

The patient’s condition and prognosis at the time of the request allowed insufficient time to obtain an FDA approval for compassionate use through the routine channels. Therefore, a second opinion supporting the administration of emergency treatment with TOL was acquired from an independent medical oncologist at a neighboring facility. All necessary ethics, IRB and legal approvals for the emergency use of TOL were obtained. Following an explanation of the potential risks and benefits of TOL treatment, informed consent for emergency use was obtained from the patient for accepting treatment and for obtaining a tissue biopsy to determine the level of VGSC expression in the tumor. Immunohistofluorescence analysis of a tumor biopsy taken from the patient’s cancer prior to the treatment revealed cellular profiles with high and low levels of VGSC labeling in a pattern similar to that observed in preclinical studies in mice and dogs that responded favorably to treatment with TOL (Figure 1). Cardiac glycoside digoxin was administered orally in the amount of 0.25 mg for four once-daily dosing cycles to achieve a therapeutic blood level (0.50–1.50 ng/mL). To maintain steady-state pharmacokinetics for treatment, a 0.25-mg dose of digoxin was administered prior to each of the 2-h periods of stimulation within a custom-built coaxial ring device (Figure 2; The Phantom Laboratory, Salem, NY, USA). The device delivered a uniform pulsed electric field (PEF; 18 V/m, 10-ms positive/negative square wave, 15-ms interstimulus interval) that has been shown to effectively activate VGSCs [3]. Treatment was administered on two successive days. 

On stimulation day 1, the patient’s height, weight and vital signs were measured, an electrocardiogram (EKG) rhythm strip was obtained, and samples of blood were collected to determine her complete blood count, serum chemistry and digoxin levels at her pretreatment baseline. The laboratory results revealed a digoxin trough level of 1.0 ng/mL and a notable hemoglobin level of 7.1 g to be monitored, but were otherwise considered acceptable. The patient was not receiving routine fluid supplementation as her hydration status as indicated by skin turgor and oral mucosal appearance was considered adequate, but due to the large tumor size, the patient also received 1 L of normal saline and 300 mg of allopurinol to reduce the risk of tumor lysis syndrome.

The patient’s intake history revealed the presence of a subclavicular indwelling power port. In consultation with the designer of the stimulation device, it was determined that it was unlikely that the port would have a negative effect on the treatment field or that the treatment field would have a negative effect on the indwelling port. Accordingly, the patient was assisted into the coaxial ring device where she adjusted her position to achieve comfortable positioning in the bore of the device on an inflatable mattress. To obviate any unanticipated adverse interaction between the PEFs and the indwelling port, test stimulation periods of 15–30 s were administered starting at 2 (the lowest field strength), 4, 6, 8, 10, 12, 14, 16 and 18 V/m (the target treatment field strength). The patient reported neither pain nor discomfort and stated that she did not perceive the pulsing electric field. PEF stimulation was then provided at 18 V/m for a total of two hours with breaks at 15-min intervals to check blood pressure and heart rate. The patient experienced no ill effects from the treatment and tolerated the procedure well. A post-treatment EKG strip and laboratory test samples were obtained and a second liter of normal saline was administered to further reduce the risk of tumor lysis syndrome. No issues of concern were observed by the staff or reported by the patient during a 1-h post-treatment period of observation. That evening, the patient experienced higher than usual levels of pain that required additional doses of her breakthrough analgesic medication, but the quality and distribution of the pain was unchanged from her pretreatment baseline. She was also noted to have a mild elevation in her temperature to 101 °F that responded to acetaminophen treatment. Urine output was maintained. As long as the increase in pain did not change in character or distribution and the reported increase in intensity was not significantly outside the range of day-to-day fluctuation that was typical for her intractable pattern and only occurred after the first day of treatment, it was thought to be related to the patient’s increased level of activity associated with travel to and from the hospital and the length of time spent in the preparation, treatment and post-treatment observation. The reported low-grade fever that responded to antipyretic treatment was a frequent nightly occurrence prior to treatment with TOL but was anticipated after TOL as an inflammatory response to the release of circulating factors after lysis and was considered a subjective indicator of effective treatment.

She returned the next morning for the second day of PEF stimulation. Pretreatment laboratory samples and an EKG rhythm strip were obtained. Her digoxin trough level was 1.1 ng/mL. As on day 1, she received a 0.25-mg dose of digoxin, a liter of normal saline and 300 mg allopurinol. The patient entered the bore of the coaxial ring device and was exposed to the PEF for a total of 2 h. Again, she tolerated the procedure well. Post-treatment laboratory tests were obtained and a liter of normal saline was administered during the post-treatment period of observation. That evening, she spiked a fever of 101.9 °F that was reduced to 100.7 °F with 1000 mg acetaminophen and returned to normal by morning. Urine output of 50 cc and 30 cc was measured twice during the night. She experienced no elevation in her pain after the second round of stimulation and was noted to be up and walking around the house “in short spurts,” more than usual. Three days after the treatment, the patient reported that she “felt better.” Her pain persisted but had decreased. She was afebrile. She noted that she had been more ambulatory and interactive, being able to carry on a reasonably long conversation, but most remarkable was that her appetite had improved significantly. She experienced two episodes of mild hemorrhagic anal discharge. This complication and hematuria were anticipated because pretreatment imaging indicated that the tumor had invaded the urinary bladder, right pelvic musculature, distal ureters and rectum and she had experienced occasional hematuria prior to treatment with TOL and was closely monitored for these potential complications. When discovered, the post-treatment hemorrhages were small, self-limited, unaccompanied by localized pain. She reported no dizziness or change in blood pressure, range: 89–102 systolic/60–63 diastolic, and her nurse reported that despite the blood loss, she had “rosy cheeks” again after three weeks of very gray pallor. The hemorrhage was not thought to be due to the damage to normal tissues but rather due to a shift of the tumor mass resulting from increased edema, inflammation or lytic necrosis.

**Figure 3:**
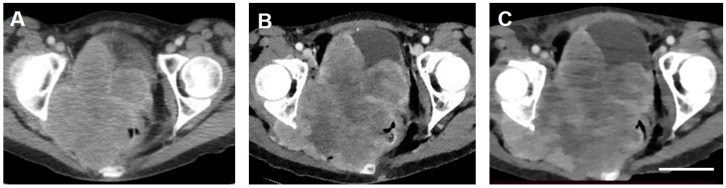
Transverse computed tomographic images of the Stage IIB carcinoma of the cervix obtained from the patient before (**A**) and 3 (**B**) and 21 days (**C**) after treatment with TOL. The images were chosen to depict cuts through the tumor at levels as closely similar as possible. Adjustments of magnifications were made to match the dimensions of bony landmarks of the femoral head and acetabulum. Adjustments of contrast and brightness for optimum clarity of the images were applied equally to all the three images. Note that the size of the tumor increases with each successive image. By contrast-enhanced CT, areas of hypodensity observed within the tumor mass appear larger and much more prominent after treatment with TOL, consistent with the reduction in measured tissue densities from 70 HU pre-treatment (**A**) to 56 HU (**B**) by day 3 and 47 HU (**C**) by day 21 post-treatment. Additional region-of-interest (ROI) reference measurements were made for each scan over the left gluteal musculature revealing values of 127 HU (**A**), 120 HU (**B**) and 125 HU (**C**), respectively. Calibration bar in C = 5 cm.

CT scans were obtained three days post-treatment and again 18 days later when the patient developed bowel obstruction requiring colectomy. Despite slight variations in the quality of imaging obtained at multiple imaging facilities, the size and density of the tumor was measured in the post-treatment scans and compared to the measurements taken from the pretreatment scan that had been obtained four weeks prior to TOL. The tumor size had increased 36% at three days post-treatment compared to the pretreatment baseline and increased another 40% 15 days later. By contrast, tumor density decreased post-treatment from 70 Hounsfield units (HU) at the baseline to 56 and 47 HU, respectively, in association with the development of large areas of hypodensity within the tumor mass occupying approximately 65–70% of its mass (Figure 3). The increase in tumor size coupled with the decrease in tumor density is consistent with a possible combination of continued tumor growth, tumor necrosis and edema and/or reactive post-treatment inflammation. The colectomy successfully relieved bowel obstruction allowing her to return home. No further treatments were permitted under the emergency use provision. The patient survived with improved quality of life for a total of nine weeks post-treatment and more than eight weeks longer than anticipated. Notably, she experienced no apparent ill effects associated with the TOL treatment.

## 3. Discussion

Targeted osmotic lysis is a fundamentally different approach to the treatment of cancer than the current treatment methods both in principle and design and is based on the observation that many cancers overexpress VGSCs and Na^+^/K^+^-ATPase [4,5,6,7]. This feature confers enhanced ability to invade normal tissues and metastasize and is found to be exceedingly prominent in advanced disease [1,8,9,10,11,12]. The TOL process takes advantage of a mechanism that is essential for survival because of its role in the maintenance of membrane potentials and cellular homeostasis. Unlike many treatment methods [13,14,15,16,17,18,19,20,21,22], TOL enhances VGSC activity, thereby greatly increasing the influx of sodium while simultaneously preventing the extrusion of these ions by blocking the sodium pumping mechanism [1,2]. The osmotic influx of water floods the cells beyond their capacity to comply, resulting in lysis of the cells [23]. Normal cells, even highly expressing excitable cells, e.g., nerve and muscle cells, are spared because sodium channel expression in normal tissues is far lower than that found in most advanced carcinomas [1], thus offering highly targeted treatment without significant morbidity.

The extension of survival beyond the expected and the lack of adverse effects that were observed in this case reflect the pattern observed in treatments of advanced carcinomas using TOL in companion animals. The decrease in tumor density evident with CT imaging in this patient is also observed in companion animals after two-day treatments with TOL. Finally, the improvement observed in clinical behavior and quality of life, evidenced by a significant improvement in appetite, increased energy and activity levels and improved behavior, is similar to clinical improvements noted in companion animals after treatment with TOL.

We have presented evidence that TOL as a standalone treatment may provide a safe, well-tolerated and effective treatment for increasing survival and stabilizing many advanced carcinomas that have typically been refractory to treatment. TOL’s positioning in the therapeutic algorithm of care or its role as a potential add-on or adjuvant to the current standard and promising new immune therapies is uncertain. As a standalone therapy, TOL might provide a means to reduce or stabilize a tumor prior to resection or to eliminate remnants of disease after removal. However, as an adjunct to treatment, it is unclear whether combining treatments might have an additive or synergistic effect, or whether therapeutic resistance induced by TOL or by the standard treatment might antagonize the effect of one treatment or the other or both. This would need to be determined.

## 4. Conclusions

Because of the highly conserved nature of the sodium channel/sodium pump mechanism, TOL has the potential to offer a safe and well-tolerated treatment for several forms of aggressive, late-stage carcinoma. This case presents evidence that TOL is associated with few adverse effects and may extend life, without compromising quality and thus warrants further study.

## 5. Patents

A patent for the technology described in this manuscript entitled Targeted Osmotic Lysis of Cancer Cells, file No. 11M01 (serial No. 13/552,909), by D.J. Paul and H.J. Gould III was allowed on 30 December 2014.

## Data Availability

The data presented in this study are available upon request from the corresponding author. The data are not publicly available but have been submitted to the FDA in accordance with the reporting policy for emergency use treatments without an IDE.
