# Correction: Gould et al. Emergency Use of Targeted Osmotic Lysis for the Treatment of a Patient with Aggressive Late-Stage Squamous Cell Carcinoma of the Cervix. *Curr. Oncol.* 2021, *28*, 2115–2122

**DOI:** 10.3390/curroncol33090535

**Published:** 2026-09-03

**Authors:** Harry J. Gould, Paige R. Miller, Samantha Edenfield, Kelly Jean Sherman, Chad K. Brady, Dennis Paul

**Affiliations:** 1 Department of Neurology, Louisiana State University Health Sciences Center, New Orleans, LA 70112, USA; 2 Oleander Medical Technologies, Baton Rouge, LA 70803, USA; pmiller@oleandermedicaltechnologies.com (P.R.M.); dpaul@lsuhsc.edu (D.P.); 3 Department of Pharmacology and Experimental Therapeutics, Louisiana State University Health Sciences Center, New Orleans, LA 70112, USA; sedenfield@sadlerhealth.org (S.E.); ksherm@lsuhsc.edu (K.J.S.); 4 Department of Radiology, West Virginia University Medical School, Morgantown, WV 26506, USA; ckbrady@hsc.wvu.edu

## Figure Legend

In the original publication [1], there was a mistake in the legend for Figure 1. The source of the primary antibody used was inadvertently omitted. The correct legend appears below. 

Figure 1. The photomicrograph (**A**) depicts the immunohistochemical labeling of VGSCs (green) in a biopsy of the Stage IIB carcinoma of the cervix obtained from the patient prior to treatment with TOL. Sections were incubated overnight with the pan-specific VGSC antibody (Alomone Labs; Jerusalem, Israel) that was pre-conjugated to an Allophycocyanin (APC) fluorophore, excitation 594 and 633 nm/emission 660 nm at a 1:200 dilution. The tissues were then processed with a goat-anti-rabbit 488 Alexa fluorophore secondary, 1:800 dilution. Nuclei were counterstained with DRAQ5 (blue; dilution 1:600). Images were taken on the Leica PMI 8 confocal microscope at 40× oil magnification. Cellular profiles were outlined, and the mean number of pixels was measured using Adobe Photoshop CS4 Extended (Adobe Systems Incorporated, San Jose, CA, USA) to quantify sodium channel expression. The histogram of the pixel counts (**B**) measures the relative level of VGSC expression in highly expressing tumor cells compared to that observed in the low level of expression observed in tumor stromal cells. A one-way ANOVA was significant (*p* < 0.00001); * = *p* < 0.001 compared to the background; ** = *p* < 0.001 compared to weakly expressing cells (Student’s *t*-test with Scheffe’s adjustment). Calibration bar in (**A**) = 25 μm. 

## Text Correction

There was an error in the original publication. A post-publication review indicated that there was an inadvertent omission of information to detail (1) how the stated estimated prognosis was obtained, (2) how it was determined that the patient was competent to make decisions on her own behalf or whether advanced directives were consulted to obtain informed consent, and (3) how the level of VGSC expression was obtained to anticipate the likelihood of a favorable response to treatment with TOL. A correction has been made to Section 2.2. Preparation, Emergency Treatment and Response, Paragraph 1. The amended text appears below.

### 2.2. Preparation, Emergency Treatment and Response

The patient’s condition and prognosis at the time of the request were drawn from the patient’s treating oncologist, who had referred her to hospice care, and an independent reviewer required by the FDA to provide an independent opinion supporting or refuting the prudence of the emergency treatment. Neither of the primary evaluators had any conflict of interest related to an affiliation with Oleander Medical Technologies. The prognostic estimate did not allow sufficient time to obtain an FDA approval for compassionate use through the routine channels. A second opinion supporting the administration of emergency treatment with TOL was acquired from an independent medical oncologist at a neighboring facility. All necessary ethical, IRB, and legal approvals for the emergency use of TOL were obtained. The patient then presented to her healthcare provider for pre-treatment evaluation. She was found to be awake and oriented, and although appearing weak, was able to follow verbal commands and respond appropriately to questions posed during physical and cognitive evaluation. Although she was deemed to be sufficiently competent to make decisions on her own behalf, she was accompanied by her husband in all aspects of the emergency use of TOL, who supported and confirmed the decisions made by the patient. Prior to requesting consideration for treatment, the patient and her husband had read about the treatment, discussed the potential risks and benefits of the procedure, and arrived at a decision to seek treatment with TOL. Following an explanation of the potential risks and benefits of TOL treatment, Informed Consent for Emergency Use was obtained from the patient for accepting treatment and for obtaining a tissue biopsy to determine the level of VGSC expression in the tumor. Immunohistofluorescence revealing VGSC expression was analyzed using ImageJ technology to measure the median pixel intensity units (PIU) of cellular profiles measured on five images taken from the patient’s biopsy sample as viewed with confocal or fluorescence microscopy. Median PIU values greater than 25 were consistent with VGSC expression sufficient to anticipate a favorable response to treatment with TOL, whereas when median PIU values were less than 15, closer to that of normal tissues, a beneficial response was considered unlikely. Pre-treatment levels of VGSC expression revealed in the patient’s biopsy sample relative to stromal cells were similar to those observed in pre-clinical trials, indicating that the patient’s cancer sufficiently over-expressed VGSCs to anticipate a favorable response to TOL treatment (Figure 1). The cardiac glycoside, 0.25 mg of digoxin, was administered orally for four once-daily dosing cycles to achieve a therapeutic blood level (0.50–1.50 ng/mL). To maintain steady-state pharmacokinetics for treatment, a 0.25 mg dose of digoxin was administered prior to each of the 2 h periods of stimulation within a custom-built coaxial ring device (Figure 2; The Phantom Laboratory, Salem, NY, USA). The device delivered a uniform pulsed electric field (PEF; 18 V/m, 10 ms positive/negative square wave, 15 ms interstimulus interval) that has been shown to effectively activate VGSCs [3]. Treatment was administered on two successive days.

The authors state that the scientific conclusions are unaffected. This correction was approved by the Academic Editor. The original publication has also been updated.
